# Biologically-Inspired Water-Swelling-Driven Fabrication of Centimeter-Level Metallic Nanogaps

**DOI:** 10.3390/mi12070735

**Published:** 2021-06-23

**Authors:** Lei Wang, Yanping Wang, Meiqin Dai, Qiuling Zhao, Xia Wang

**Affiliations:** Shandong Advanced Optoelectronic Materials and Technologies Engineering Laboratory, College of Mathematics and Physics, Qingdao University of Science and Technology, Qingdao 266061, China; a17854296575@163.com (Y.W.); daimeiq@163.com (M.D.); sdqlzhao@163.com (Q.Z.)

**Keywords:** water-swelling-driven fabrication, metallic nanogap, large-area, low-cost

## Abstract

Metallic nanogaps have great values in plasmonics devices. However, large-area and low-cost fabrication of such nanogaps is still a huge obstacle, hindering their practical use. In this work, inspired by the cracking behavior of the tomato skin, a water-swelling-driven fabrication method is developed. An Au thinfilm is deposited on a super absorbent polymer (SAP) layer. Once the SAP layer absorbs water and swells, gaps will be created on the surface of the Au thinfilm at a centimeter-scale. Further experimentation indicates that such Au gaps can enhance the Raman scattering signal. In principle, the water-swelling-driven fabrication route can also create gaps on other metallic film and even nonmetallic film in a low-cost way.

## 1. Introduction

Metallic gaps with tens of nanometers or several nanometers in size possess unique physical and chemical properties, which have attracted enormous attention in plasmonics devices [1,2,3,4,5], transistors [6,7,8,9] and molecular electronics [10,11]. Typically, the metallic nanogap can generate surface plasma resonance that will strongly enhance the localized electromagnetic field. This effect has great prospects in SERS (surface enhanced Raman scattering) [12,13,14,15] and sensing [16,17,18]. In these application scenarios, fabricating large-area metallic nanogaps in a low-cost manner is of great importance.

At present, a variety of techniques have been developed to fabricate independent or arrayed nanogaps. For instance, resist-based lithography such as high-resolution EBL (electron beam lithography) is able to directly fabricate arrays of nanosized gaps [19,20,21]. In addition, FIB (focused ion beam etching) has been reported as a resist-free technique for fabricating arbitrarily designed metallic nanogaps [22]. However, fabricating large-area metallic nanogaps using EBL and FIB is very expensive. Chemical synthesis of internal metallic nanogaps have been reported [23], but the large-area preparation is still an obstacle. Moreover, nanosphere lithography have been used to fabricate plasmonics nanogap structures [24,25,26], but the structure density and the control of large-area preparation need to be further improved. More effective and new technologies are worth exploring to fabricate large-area metallic nanogaps in a low-cost manner.

In fact, abundant deformation phenomena have been already existed in nature for millions of years, including wrinkling [27] and dewetting [28], which have inspired researchers to study mechanical properties of micro-nano materials [29,30,31] and establish smart ways for building micro-nanostructures [32,33]. In this work, the authors were inspired by the cracking behavior of the tomato skin after raining and then a water-swelling-driven fabrication route was developed to create metallic nanogaps at a centimeter-scale. This biologically-inspired fabrication method is low-cost and high-efficiency.

## 2. Materials and Methods

A 0.17 mm thick glass substrate was cleaned by acetone, alcohol, deionized water, respectively and then dried by N_2_. Afterwards, Super Absorbent Resin (SAP) powders (the main component is sodium polyacrylate) were uniformly pasted on the glass substrate via a double sticky tape. Then, a 100 nm thick Au film was deposited onto the SAP layer with a simple ion sputter, which forms a double-layered Au/SAP structure. The deposition time was 200 s and the deposition current was 10 mA and the deposition air pressure was 8 Pa. After dropping deionized water onto the Au/SAP double-layers, Au-Gap/SAP structure was obtained.

The optical images were obtained with an optical microscope (Olympus, BX51, Tokyo, Japan). The reflectance spectrum was measured with a spectrograph (Ocean optics USB4000, Orlando, FL, USA). Surface morphologies were characterized with a scanning electron microscope (SEM) (ZEISS sigma 300, Jena, Germany) operated at 5 kV.

Rhodamine 6g powders were uniformly placed on the large-area Au/SAP structures and Au-Gap/SAP structures, respectively, where the average number density of the Rhodamine 6g molecules is about 10^5^/μm^2^. A microscopic Raman Spectrometer (Renishaw inVia, Beijing, China) was used to measure the Raman scattering signals at randomly-selected ten locations, respectively. The measurements were conducted at room temperature using the excitation laser with a wavelength of 785 nm, laser spot radius of ~3 μm, laser power of 0.5 mw and the integration time of 10 s for each Raman spectrum measurement.

## 3. Results

**Figure 1:**
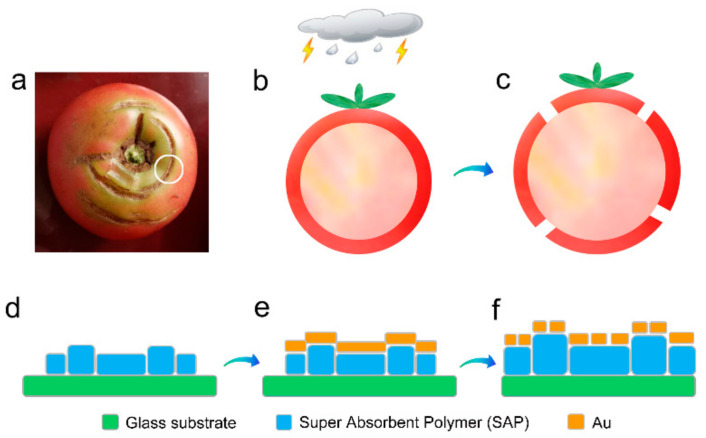
(**a**) Cracks on the tomato skin after raining (marked with the white circle). (**b**,**c**) Simple formation mechanism of the cracks on the tomato skin. (**d**–**f**) Schematic diagram of the water-swelling-driven route to fabricate Au nanogaps.

As shown in Figure 1a, after rain, cracks always appear on the tomato skin, which is generally harmful for the crop production. This phenomenon is easy to explain. As shown in Figure 1b,c, the internal pulp of the tomato absorbs a large amount of water after rain and then swells. Finally, the tomato skin splits open. This simple natural phenomenon inspires us to develop a water-swelling-driven route to fabricate small metallic gaps, which has significant advantage in large-area and low-cost manufacturing.

According to the above idea, the fabrication process of the Au gaps is designed as described in the schematic diagrams (Figure 1d–f). Super Absorbent Polymer (SAP) powders can be chosen as the water-swelling material. After the Au/SAP double layers (Figure 1e) touch water, the SAP layer swells and gaps form on the surface Au film (Figure 1f). This preparation process does not require any expensive nanofabrication technologies such as EBL and FIB.

**Figure 2:**
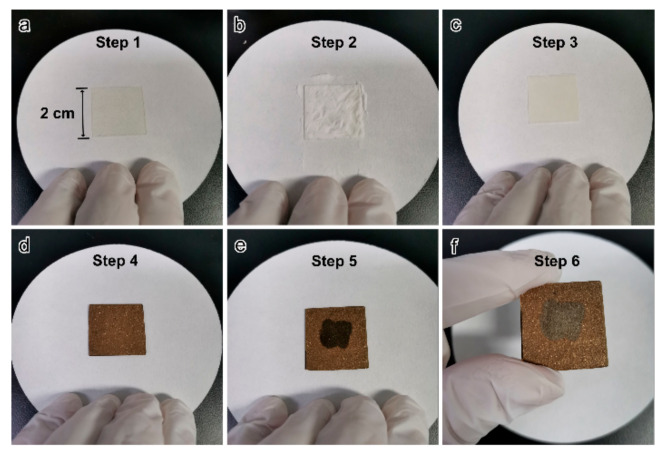
(**a**) A double sticky tape is pasted on a glass substrate. (**b**,**c**) Preparation of the SAP layer on the substrate. (**d**) Deposition of the Au thin film on the SAP layer. (**e**,**f**) Dropping deionized water onto the Au film to create the Au nanogaps.

The detailed preparation process is shown in Figure 2. In step 1, a double sticky tape is pasted on a 0.17 mm thick glass substrate (Figure 2a). In step 2, a mass of SAP powders are spread over the tape (Figure 2b). In step 3, most of the SAP powders are blown off the substrate by the pure nitrogen flow. Finally, only a thin layer of SAP powders that have been sticked firmly on the tape are left on the substrate (Figure 2c). The thickness of the SAP powder layer is about tens of micrometers, which is close to a single layer or a few layers stacking due to the variation in SAP powder size. In step 4, about 100 nm thick Au film is deposited on the SAP layer (Figure 2d) by the low-cost ion sputtering method. In step 5, 2 μL of deionized water is dropped on the surface of Au film for each time at different positions and the total water volume we dropped is 20 μL (Figure 2e). In step 6, after natural drying for a few minutes, the Au nanogaps are finally created in the middle (gray) area (Figure 2f). It should be noted that the entire preparation process is fast, simple and inexpensive. In addition, the Au nanogaps distributed over the centimeter-level area.

**Figure 3:**
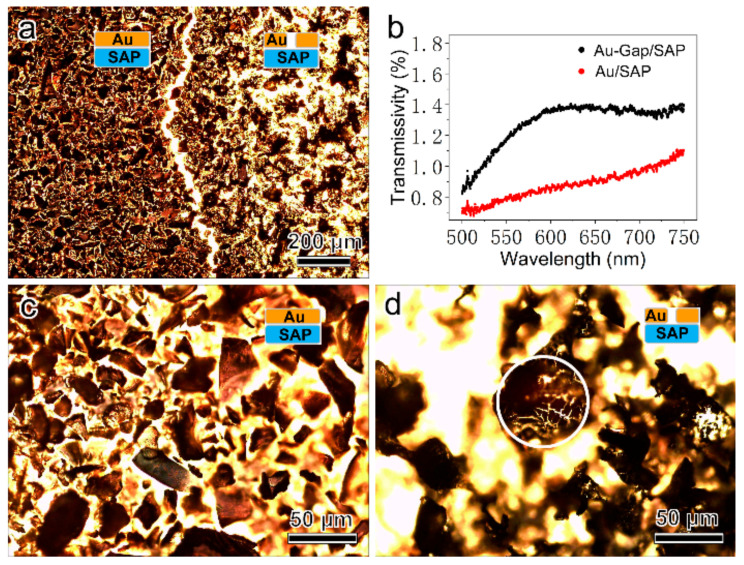
(**a**) Optical photograph of the Au/SAP structures (left side) and the Au-Gap/SAP structures (right side). (**b**) Visible transmission spectrum of the Au/SAP structures (red) and the Au-Gap/SAP structures (black). (**c**) Optical photograph of the Au/SAP structures. (**d**) Optical photograph of the Au-Gap/SAP structures (marked with the white circle).

In order to investigate the Au nanogaps in detail, transmission-type optical characterization was implemented. The left side area in Figure 3a shows the Au film deposited on the SAP layer (i.e., Au/SAP structures). The right side area in Figure 3a is where the deionized water dropped on, which creates Au nanogaps on the surface (i.e., Au-Gap/SAP structures). Clearly, as shown in Figure 3a, the right side area has larger transmission light intensity due to the existence of vast Au gaps, which has been also verified by the visible transmission spectrum (Figure 3b). As shown in Figure 3c and Figure S1a, before dropping deionized water, the Au film deposited on the SAP powders is continuous. However, after dropping deionized water, as shown in Figure 3d and Figure S1b, cracks can be apparently observed on the Au film.

**Figure 4:**
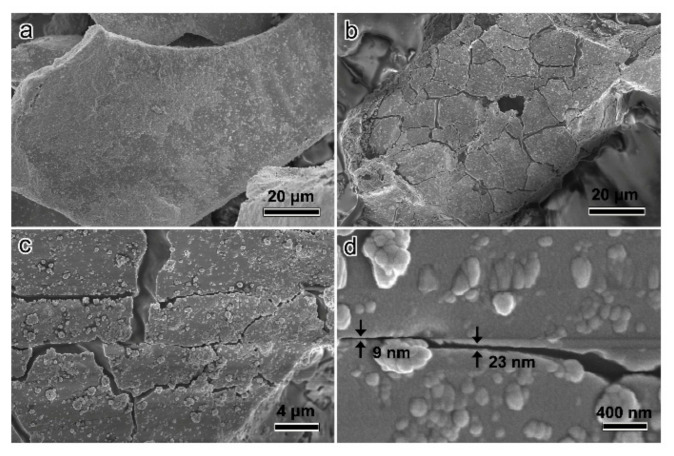
(**a**) SEM characterization of the Au/SAP structures before dropping deionized water. (**b**–**d**) SEM characterization of the Au Gaps obtained by the water-swelling-driven fabrication.

Furthermore, SEM characterization was implemented to investigate the morphology and size of Au nanogaps. As shown in Figure 4a, without dropping deionized water, the Au film deposited on the SAP powder has no cracks. After dropping deionized water, as shown in Figure 4b–d, the SAP powders swell and vast cracks appear on the surface Au film. The diameter of most Au islands is about several micrometers. The width of Au gaps has a distribution from several nanometers to hundreds of nanometers and the statistical distribution of 100 randomly selected Au gaps is shown in Figure S2. As shown in Figure 4d, the fabricated Au nanogap can be as narrow as 9 nm.

**Figure 5:**
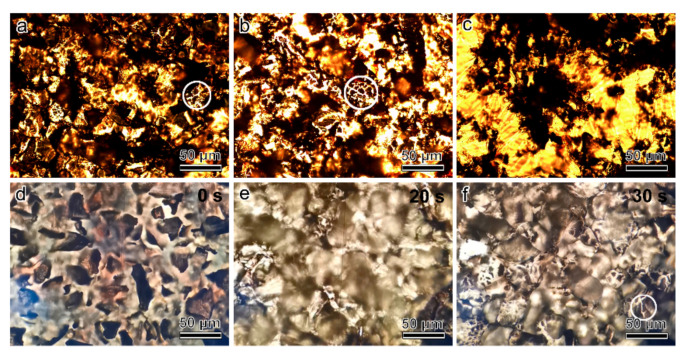
(**a**) Optical photograph of the Au-Gap/SAP structures (marked with the white circle) where the Au film thickness is 100 nm and the deionized water dropped is 2 μL. (**b**) Optical photograph of the Au-Gap/SAP structures (marked with the white circle) where the Au film thickness is 200 nm and the deionized water dropped is 2 μL. (**c**) Optical photograph of the Au-Gap/SAP structures where the Au film thickness is 200 nm and the deionized water dropped is 20 μL. (**d**) Video snapshot before dropping deionized water. (**e**) Video snapshot when dropping 5 μL deionized water. (**f**) Video snapshot after dropping deionized water for about 10 s and the Au-Gap/SAP structures are marked with the white circle.

In addition, different influential factors of gap generation were further investigated including the thickness of Au film, the water volume dropped each time and the time variation. Firstly, it was found that the Au nanogaps could be obtained for a wide range of the Au film thickness from 100 nm to 200 nm (Figure 5a,b and Figure S3a,b). Secondly, the suitable deionized water volume dropped each time is smaller than a few microliter (Figure 5a,b). If too much water is dropped onto the Au film, the SAP layer will be overinflated and Au nanogaps can not be obtained (Figure 5c). Thirdly, the Supplementary Video S1 shows the dynamic formation process of the Au gaps when dropping deionized water and the Au gaps can form within about 10 s (Figure 5d–f and Figure S3c).

In order to show the possible application of the water-swelling-driven Au nanogaps, we have experimentally confirmed that the Au-Gap/SAP structures have better SERS effect than the as-deposited Au/SAP structures using Rhodamine 6g molecules (Figure S4).

## 4. Discussion

The biologically-inspired water-swelling-driven fabrication strategy has huge advantage in large-area and low-cost manufacturing of nanogaps. In this paper, the SAP powder as one kind of water-swelling material was just used to provide a demonstration of proof of concept. In fact, in addition to the large-area and low-cost advantages, the nanogaps might be possible delicately controlled if more homogeneous water-swelling materials are applied.

In addition, according to the water-swelling-driven fabrication mechanism, the Au film can be distinctly replaced by other metallic and even nonmetallic film, which will greatly expand the scope of its application.

## 5. Conclusions

Large-area and low-cost fabrication of metallic nanogaps is a huge obstacle, hindering their practical use. In this work, inspired by the cracking behavior of the tomato skin, a water-swelling-driven fabrication route is developed to manufacture centimeter-scale Au nanogaps in a low-cost manner. In addition, the large-area Au-Gap structures have experimentally shown a SERS effect. In future, the nanogaps might be delicately controlled if more homogeneous water-swelling materials are applied. Moreover, in principle, Au film can be replaced by other metallic and even nonmetallic film, which will greatly expand the application scope of the nanogaps.

## Supplementary Materials

The following are available online at https://www.mdpi.com/article/10.3390/mi12070735/s1, Figure S1: SEM characterization results. Figure S2: Statistical size distribution of 100 randomly-selected Au gaps. Figure S3: SERS effect of the Au-Gap/SAP structures. Figure S4: SEM characterization results of the gap structures. Video S1: Dynamic formation process of the Au gaps when dropping deionized water.
