# Supplementary material for: Biologically-Inspired Water-Swelling-Driven Fabrication of Centimeter-Level Metallic Nanogaps
Source: Micromachines (Basel). 2021 Jun 23;12(7):735. doi: 10.3390/mi12070735 (PMC8305456; doi:10.3390/mi12070735)
Supplement: Supplementary file 1 [file micromachines-12-00735-s001.zip › Supporting information micromachines-1162316.pdf]

Supporting Information

Biologically-inspired water-swelling-driven fabrication of centimetre-level metallic nanogaps

Lei Wang *, Yanping Wang, Meiqin Dai, Qiuling Zhao and Xia Wang *

Shandong Advanced Optoelectronic Materials and Technologies Engineering Laboratory, College of Mathematics and Physics, QingDao University of Science and Technology, Qingdao, China; a17854296575@163.com (Y.W.); daimeiq@163.com (M.D.); sdqlzhao@163.com (Q.Z.)
*Correspondence: phdwangl@163.com; phwangxia@163.com

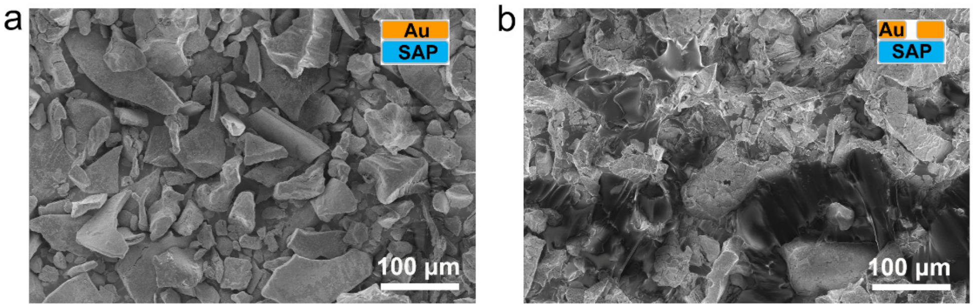

Figure S1. SEM characterization results. (**a**) SEM image of the Au/SAP structures before dropping deionized water. (**b**) SEM image of the Au-Gap/SAP structures after dropping deionized water.

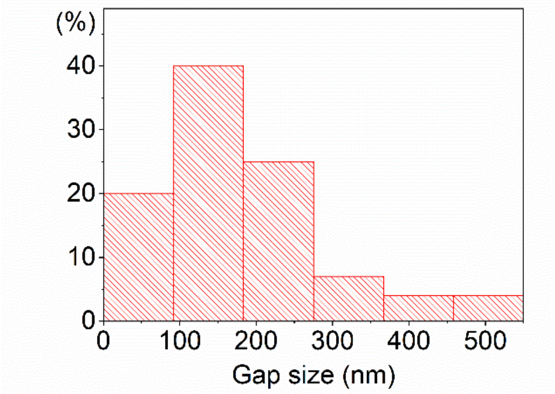

Figure S2. Statistical size distribution of 100 randomly-selected Au gaps.

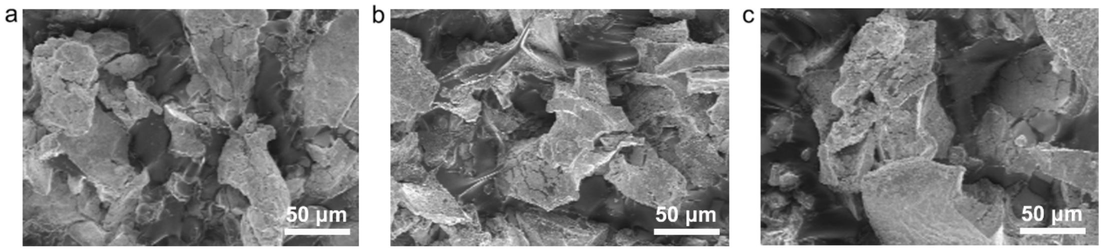

Figure S3. SEM characterization results of the gap structures. (**a**) SEM image corresponding to Figure 5a. (**b**) SEM image corresponding to Figure 5b. (**c**) SEM image corresponding to Figure 5f.

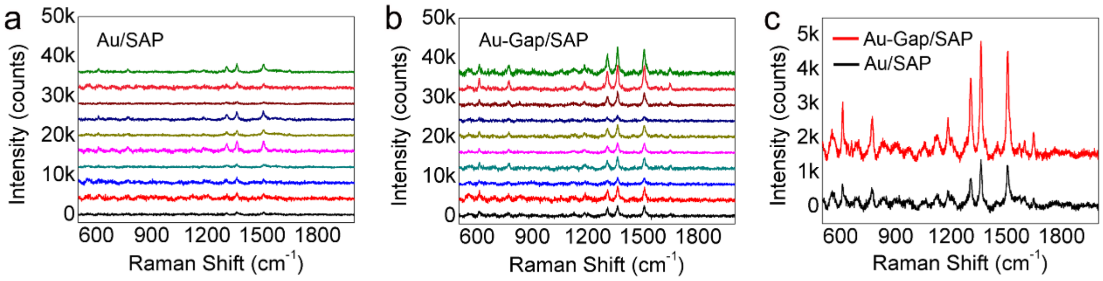

Figure S4. SERS effect of the Au-Gap/SAP structures. (**a**) SERS spectrums of R6g molecules randomly collected from 10 different sites on the as-deposited large-area Au/SAP structures. (**b**) SERS spectrums of R6g molecules randomly collected from 10 different sites on the large-area water-swelling-driven Au-Gap/SAP structures. (**c**) Average spectral intensity of the 10 SERS spectrums shown in a and b, respectively.
